# Supplementary material for: The slowest timescales of neural synchronization reveal the strongest influence of auditory distraction
Source: Front Hum Neurosci. 2025 Sep 4;19:1623431. doi: 10.3389/fnhum.2025.1623431 (PMC12445148; doi:10.3389/fnhum.2025.1623431)
Supplement: Supplementary file 12 [file Data_Sheet_1.docx]

Supplementary Material

# Supplementary Data

Exemplar audio files are included as supplementary data. Descriptions of the included files:

Audio 1.WAV An example target random-pattern stimulus used in the EEG sessions, corresponding to the illustration in Figure 1C.

Audio 2.WAV An example target random-random stimulus used in the EEG sessions.

Audio 3.WAV An example target pattern stimulus used in the at-home testing, corresponding to the illustration in Figure 2B.

Audio 4.WAV An example melodic distractor stimulus corresponding to the top panel in Figure 2A and in Figure 2B

Audio 5.WAV An example melodic distractor stimulus corresponding to the middle panel in Figure 2A

Audio 6.WAV An example melodic distractor stimulus corresponding to the bottom panel in Figure 2A

Audio 7.WAV An example noise distractor stimulus corresponding to the illustration in Figure 4F, and matched to Audio 4.WAV

Audio 8.WAV An example noise distractor stimulus matched to Audio 5.WAV

Audio 9.WAV An example noise distractor stimulus matched to Audio 6.WAV

Audio 10.WAV An example pattern target presented with a melodic distractor as used in the at-home testing

Audio 11.WAV An example pattern target presented with a matched noise distractor as used in the at-home testing
